# Isoprenoids responsible for protein prenylation modulate the biological effects of statins on pancreatic cancer cells

**DOI:** 10.1186/s12944-017-0641-0

**Published:** 2017-12-20

**Authors:** Helena Gbelcová, Silvie Rimpelová, Zdeněk Knejzlík, Jana Šáchová, Michal Kolář, Hynek Strnad, Vanda Repiská, Walter Cosimo D’Acunto, Tomáš Ruml, Libor Vítek

**Affiliations:** 1 Institute of Medical Biology, Genetics and Clinical Genetics, Faculty of Medicine, Comenius University, Bratislava, Slovakia; 2 Department of Biochemistry and Microbiology, University of Chemistry and Technology, Prague, Czech Republic; 3 Laboratory of Genomics and Bioinformatics, Institute of Molecular Genetics of the Academy of Sciences of the Czech Republic, Prague, Czech Republic; 4 Institute of Medical Biochemistry and Laboratory Diagnostics, and 4th Department of Internal Medicine, 1st Faculty of Medicine, Charles University, Prague, Czech Republic

**Keywords:** Farnesyl pyrophosphate, Gene expression, Geranylgeranyl pyrophosphate, HMG-CoA reductase inhibitors, Isoprenoids, *K-Ras* oncogene, Mevalonate, Pancreatic cancer, Prenylation, Statins

## Abstract

**Background:**

Statin treatment of hypercholesterolemia is accompanied also with depletion of the mevalonate intermediates, including farnesyl pyrophosphate (FPP) and geranylgeranyl pyrophosphate (GGPP) necessary for proper function of small GTPases. These include Ras proteins, prevalently mutated in pancreatic cancer. In our study, we evaluated the effect of three key intermediates of the mevalonate pathway on GFP-K-Ras protein localization and the gene expression profile in pancreatic cancer cells after exposure to individual statins.

**Methods:**

These effects were tested on MiaPaCa-2 human pancreatic cancer cells carrying a K-Ras activating mutation (G12C) after exposure to individual statins (20 μM). The effect of statins (atorvastatin, lovastatin, simvastatin, fluvastatin, cerivastatin, rosuvastatin, and pitavastatin) and mevalonate intermediates on GFP-K-Ras protein translocation was analyzed using fluorescence microscopy. The changes in gene expression induced in MiaPaCa-2 cells treated with simvastatin, FPP, GGPP, and their combinations with simvastatin were examined by whole genome DNA microarray analysis.

**Results:**

All tested statins efficiently inhibited K-Ras protein trafficking from cytoplasm to the cell membrane of the MiaPaCa-2 cells. The inhibitory effect of statins on GFP-K-Ras protein trafficking was partially prevented by addition of any of the mevalonate pathway’s intermediates tested. Expressions of genes involved in metabolic and signaling pathways modulated by simvastatin treatment was normalized by the concurrent addition of FPP or GGPP. K-Ras protein trafficking within the pancreatic cancer cells is effectively inhibited by the majority of statins; the inhibition is eliminated by isoprenoid intermediates of the mevalonate pathway.

**Conclusions:**

Our data indicate that the anticancer effects of statins observed in numerous studies to a large extent are mediated through isoprenoid intermediates of the mevalonate pathway, as they influence expression of genes involved in multiple intracellular pathways.

**Electronic supplementary material:**

The online version of this article (10.1186/s12944-017-0641-0) contains supplementary material, which is available to authorized users.

## Background

World-wide, statins are prescribed as hypocholesterolemic drugs preventing cardiovascular morbidity as well as mortality [1]. They lower cholesterol by inhibiting HMG-CoA reductase, the rate-limiting enzyme of the mevalonate pathway for de novo synthesis of cholesterol. However, the statins’ inhibitory action on HMG-CoA reductase also results in depletion of the downstream intermediate products of this pathway, such as farnesyl pyrophosphate (FPP) and geranylgeranyl pyrophosphate (GGPP), which modify and target small GTPases, such as Ras proteins, to their site of action [2]. Farnesylation is also required for Ras protein anchoring in the plasma membrane, a compartment crucial for its proper signaling function [3]. Accordingly, the inhibition of prenylation (farnesylation and geranylgeranylation), became a plausible approach to modify tumor cell proliferation [4], but also seems to have therapeutic potential for cardiovascular and pulmonary medicine [5]. Importantly, in healthy cells, cholesterol and exogenous isoprenoids suppress HMG-CoA reductase via post-translational downregulation [6]. On the contrary, tumor cells are resistant to cholesterol-mediated suppression, although they remain sensitive to dietary, tumor-specific isoprenoid-mediated suppression of mevalonate synthesis [7]. The K-Ras signaling pathway is of special importance in pancreatic cancer, since >90% of these tumors bear activating mutations in *K-Ras* oncogene [8]. This oncogenic signaling pathway in pancreatic cancer is complex and passes through three major effector branches: Raf/Mek/Erk, PI3K/Pdk1/Akt and the Ral guanine nucleotide exchange factor pathway [9]. Because of this widespread effects, the K-Ras signaling pathway is a promising target in pancreatic cancer and various novel therapeutic approaches to suppress K-Ras signaling are currently under investigation. However, despite these comprehensive efforts, effective anti-Ras drugs have yet to reach the clinic [10].

Recently, we demonstrated substantial differences in the antitumor effects of individual statins used in clinical practice in an experimental model of human pancreatic cancer [11]. The effect of statins on the viability of pancreatic cells was, at least partially, caused by impairment of K-Ras protein trafficking, since all statins except for pravastatin efficiently inhibited GFP-K-Ras protein translocation from cytoplasm to the cell membrane [11].

Hence, the primary aim of the present study was to compare the effect of the mevalonate pathway intermediates on GFP-K-Ras protein dislocation in the pancreatic cancer cells treated with individual statins, and assess the role of these intermediates in the tumor-suppressive action of statins. The observations that addition of FPP or GGPP rescued the viability of cancer cells treated with statins [11] suggested that the effects of these compounds on gene expression changes were induced by statins. Thus, we also aimed to screen for differential gene expression of pancreatic cancer cells exposed to simvastatin, and to assess the gene expression profile after simultaneous exposure of these cells to FPP and GGPP in the whole genome expression array.

## Methods

### Materials

In each of the experiments, pure forms of the following statins were used: atorvastatin, lovastatin, simvastatin, fluvastatin, cerivastatin, rosuvastatin, and pitavastatin (all obtained from Alexis; San Diego, CA, USA); mevalonate, FPP, and GGPP were purchased from Sigma (St. Louis, MO, USA).

### Cell cultures

The human pancreatic cancer cell line MiaPaCa-2 (ATCC, Manassas,VA, USA) carrying the activation mutation in the *K-Ras* oncogene (G12C) was maintained in the exponential phase of growth in a humidified atmosphere containing 5% CO_2_ at 37 °C, in DMEM medium supplemented with 10% fetal bovine serum. The final concentration of methanol, which was used for dissolving the experimental compounds, was below 1%. The cell line was authenticated at ATCC by STR profiling before distribution, and also re-authenticated by the end of the study by an external laboratory (Generi Biotech, Hradec Kralove, Czech Republic).

### Transfection and imaging of GFP-K-Ras intracellular localization

MiaPaCa-2 cells were seeded in 6-well cell culture plates with sterile glass coverslips 5 h before transfection. Transfection with pEGFP-K-Ras plasmids, prepared as previously described [11], was carried out using FuGene® 6 (Roche, Basel, Switzerland) according to the manufacturer’s instructions. After 24 h of incubation, the medium was changed, and the tested compounds were added: statins to a final concentration of 20 μM, mevalonate to a final concentration of 600 μM, and FPP or GGPP to final concentrations of 17 μM. All experiments were performed in triplicates and repeated 4 times for all measurements.

The tested concentrations were chosen based on our previously published data [11], in which all the statins tested at the 20 μM concentration efficiently inhibited GFP-K-Ras protein accumulation in the plasma membrane, while FPP and GGPP at 17 μM substantially abrogated the tumor cell growth inhibitory effect of all statins tested in vitro. Simultaneously, mevalonate at the concentration of 600 μM completely eliminated the cytotoxic effect of all of the statins tested in vitro, whereas the equimolar concentration of mevalonate (17 μM) only had a partial effect [11].

After an additional 24 h, the cells were washed with phosphate buffered saline (PBS) and fixed with 4% formaldehyde in PBS for 20 min. Intracellular localization of GFP-K-Ras protein was visualized by fluorescence microscopy, using an Olympus IX-81 microscope and processing with Olympus xcellence RT software (Olympus, Tokyo, Japan).

### DNA microarray analysis

The effects of 12 μM concentrations of simvastatin (corresponding to its IC_50_ value after 24 h [11]), and 17 μM FPP or GGPP on MiaPaCa-2 pancreatic cancer cell gene expression were investigated 24 h post-inoculation; the cells treated with vehicle (methanol <1%) served as control. Simvastatin was selected based on our previous study as the statin most efficiently inhibiting pancreatic cancer out of the clinically used statins [11].

The cells from two parallel cultures (10 cm^2^ culture dishes) were lysed in the stage of subconfluency using the RLT lysis buffer supplied in a RNeasy Mini Kit (Qiagen, USA). Total RNA was isolated by RNeasy Micro Kit (QIAGEN, USA) according to the procedure for animal cells. The quantity of RNA was measured by a NanoDrop ND-1000 spectrophotometer (NanoDrop Technologies LLC, USA). The quality of the RNA was analyzed by an Agilent 2100 Bioanalyser (Agilent Technologies, CA, USA). Those RNA samples that had a RIN (RNA integrity number) above 9 were used for further analysis.

An Illumina HumanWG-6_V3 chip (Illumina, USA) was used for the microarray analysis following the standard protocol. Total RNA (150 ng) was amplified using an Illumina TotalPrep RNA Amplification Kit (Ambion, USA), and 1.5 μg of the amplified RNA was hybridized on the chips according to the manufacturer’s protocol. The analysis was performed in two biological replicates for the FPP, GGPP, and combination treatments, in four biological replicates for the simvastatin treatment, and in eight biological replicates for the control group.

The raw data was preprocessed using GenomeStudio software (version 1.9.0.24624; Illumina, CA, USA), with the beadarray [12] and the limma package [13] of the Bioconductor [14] within the R environment [15]: the transcription profiles were background corrected using a normal-exponential model, quantile normalized, and variance stabilized using base 2 logarithmic transformation. A moderated t-test was used to detect differentially expressed transcripts (within limma). Storey’s q-value (q < 0.05; [16]) and a minimally twofold change in expression intensity were required for significantly differentially transcribed genes. Batch effects were accounted for in the statistical model in limma, and eliminated for visualization purposes. The MIAME compliant data has been deposited to the ArrayExpress database (accession number E-MTAB-3263).

Gene set enrichment analysis (GSEA) was performed using the method of Tian et al. [17], and Fisher’s exact test on KEGG pathways [18]. We required statistical significance in both tests, with a false discovery rate of FDR < 0.001 in the more sensitive test of Tian, and FDR < 0.05 in Fisher’s exact test.

### Quantitative real-time PCR

The genes for quantitative real-time PCR analysis were selected to validate DEG and their possible effect on KEGG pathways affected by simvastatin, FPP, and GGPP treatment. This analysis also served to validate changes detected by the microarray technology in general. The RNA derived from the same pool as for microarray experiments was used for quantitative real-time PCR analyses. Reverse transcription was performed with a QuantiTect Reverse Transcription Kit (QIAGEN Inc., USA). The RT-PCR was performed with a LightCycler 2.0 System using a LightCycler 480 DNA SYBR Green I Master kit (Roche Diagnostics, Germany), with the results analyzed by the LightCycler software. The resulting crossing point values were normalized using reference genes RPS9, TBP, and GAPDH. Relative fold changes of expression intensity in statin-treated against control samples were computed under assumption of perfect effectivity of PCR amplification. Statistical significance was estimated using Student’s *t*-test. All computations were performed within the R environment [15]. The list of amplicons/primers of target and housekeeping genes are provided in Additional file 1: Table S1.

## Results

### The effect of mevalonate, FPP, and GGPP on statin-mediated GFP-K-Ras protein translocation in the MiaPaCa-2 human pancreatic cancer cells

**Fig. 1:**
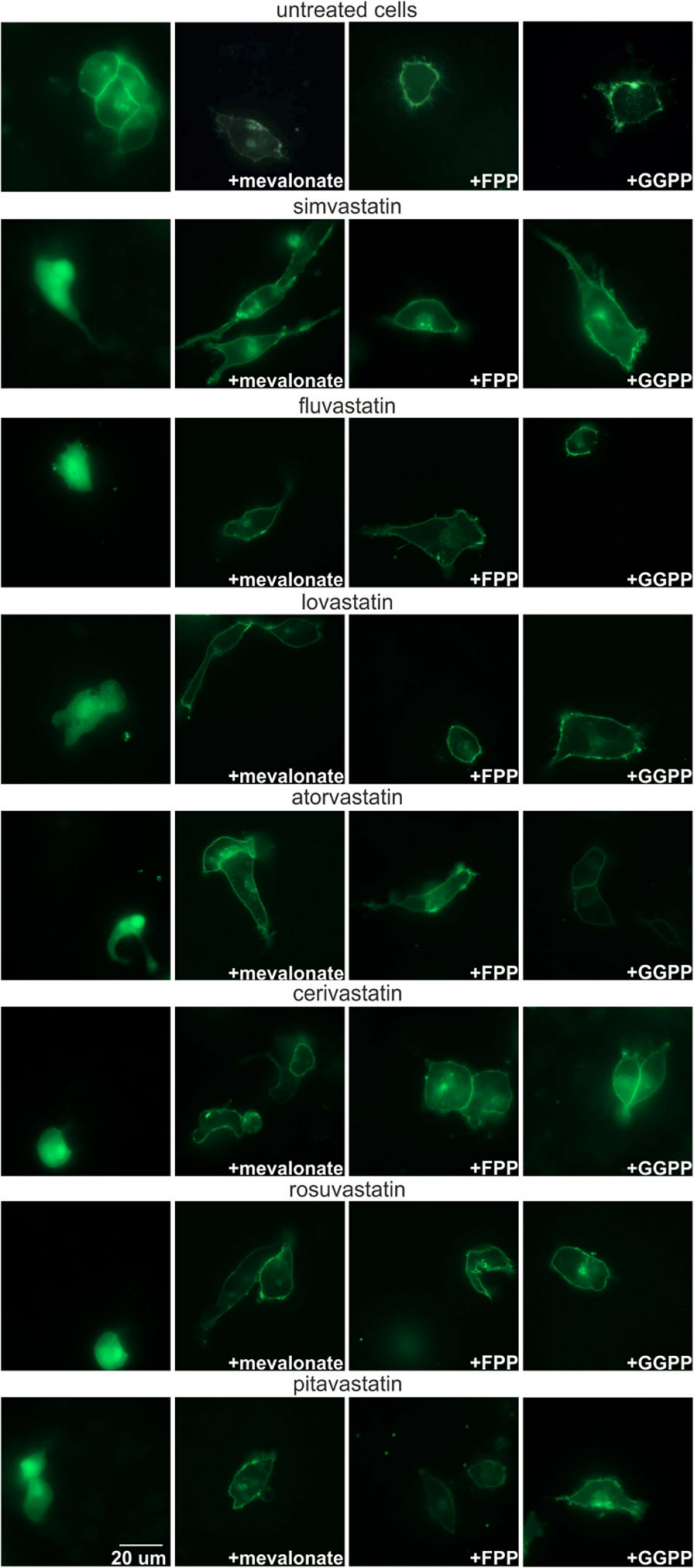
The effect of mevalonate (column B), FPP (column C), and GGPP (column D) on statin-mediated GFP-K-Ras protein translocation in the human pancreatic cancer cells. MiaPaCa-2 human pancreatic cancer cells expressing GFP-K-Ras were treated with statins (20 μM) together with mevalonate (600 μM), FPP (17 μM) or GGPP (17 μM), respectively, for 24 h. MiaPaCa-2 cells treated only with statins (column A) were used as the control of the statins’ effect on GFP-K-Ras protein localization. Simva = simvastatin; FPP = farnesyl pyrophosphate; GGPP = geranylgeranyl pyrophosphate

In our current study, we have confirmed the inhibitory effect of all clinically used statins on K-Ras protein accumulation in the cell plasma membrane (Fig. 1, column A). Pravastatin was not tested, since it failed to inhibit the GFP-K-Ras protein trafficking from the cytoplasm to the plasma membrane in our previous study [11]. This statin-mediated action was completely eliminated by an excess of mevalonate (Fig. 1, column B), indicating that this biologically important effect of statins is due to the inhibition of the mevalonate pathway.

This observation was also corroborated by the same eliminating effects mediated by both FPP and GGPP even at much smaller concentrations (17 μM), although the trafficking of the GFP-K-Ras protein to the plasma membrane was not rescued in all of the treated cells (Fig. 1, columns C and D). We did not observe any apparent differences in these effects between both isoprenoid compounds, confirming the aforementioned statement that all of the tested statins affected the GFP-K-Ras protein translocation due to isoprenoids depletion caused by HMG-CoA reductase inhibition.

### The effect of FPP and GGPP on simvastatin-mediated changes in the gene expression of MiaPaCa-2 human pancreatic cancer cells

**Fig. 2:**
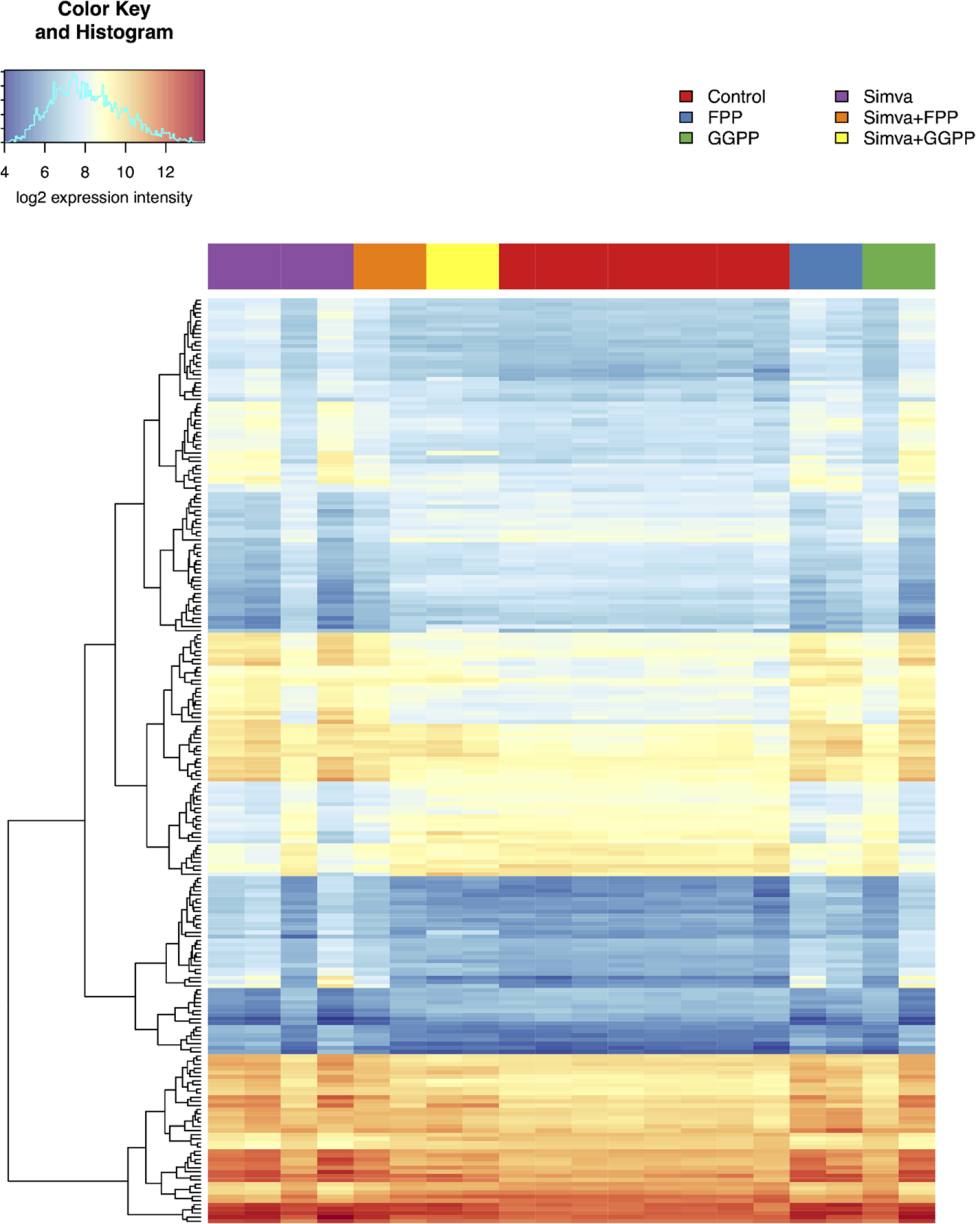
Heatmap illustrating log2-expression intensities of the genes that are differentially expressed after treatment of MiaPaCa-2 cells with simvastatin, FPP, GGPP, or their combinations. Only the differentially transcribed genes with at least two-fold change in expression intensity and q < 0.05 are shown. The list and sequence of the genes correspond to those stated in the Additional file 1: Table S4. Simva = simvastatin; FPP = farnesyl pyrophosphate; GGPP = geranylgeranyl pyrophosphate

Overall numbers of genes with significant changes in expression after simvastatin, FPP, or GGPP treatment of MiaPaCa-2 human pancreatic cancer cells are listed in Additional file 2: Table S2. There were about 200 genes affected by the simvastatin treatment. The changes in gene expression caused by simvastatin were eliminated by simultaneous addition of FPP with simvastatin, and the gene expression profile of the cells treated concurrently with simvastatin and FPP resembled control untreated cells (Fig. 2). In particular, we observed normalization of expression of KRAS, which is directly related to pancreatic cancer progression, and mevalonate pathway related genes (HMGCS1, MVD, MVK, and PDSS1). FPP per se influenced gene expression of the cells and the genes, whose expression was the most significantly affected by FPP treatment, were largely identical to those affected by the simvastatin treatment. We observed changes in the genes involved in DNA replication (*POLA2*, *POLD4*, *PRIM1*), RAS signaling related genes (*RRAS*, *RHOB*, *RAB17*), and genes of the mevalonate pathway (*MVD*, *HMGCS1*, *HMGCR*). However, the number of the genes affected by FPP was smaller and their change of expression weaker. GGPP alone did not affect gene expression of MiaPaCa-2 human pancreatic cancer cells; nevertheless, it eliminated almost all the changes in gene expression caused by simvastatin when the cells were treated simultaneously with GGPP and simvastatin (Table 1).

**Table 1:** Genes with significantly changed expression after simvastatin, FPP, and GGPP treatment of MiaPaCa-2 human pancreatic cancer cells

	Simva (S)	FPP (F)	Overlap (S) and (F)	Simva + FPP	GGPP	Simva + GGPP (SG)	Overlap (S) and (SG)
Upregulated genes (No.)	127	56	34	0	0	5	3
Downregulated genes (No.)	72	14	12	0	0	0	0
Differentially expressed genes (No.)	199	70	46	0	0	5	3

Number of genes differentially transcribed between samples treated with simvastatin (12 μM), FPP (17 μM) or GGPP (17 μM) compared to control samples of untreated cells, as well as for each combination of simvastatin and an inhibitor. Only the transcripts with a minimally two-fold change in expression intensity and q < 0.05 were counted. See Fig. 2 and Additional file 1: Table S4 for more details

*Simva* simvastatin, *FPP* farnesyl pyrophosphate, *GGPP* geranylgeranyl pyrophosphate

**Fig. 3:**
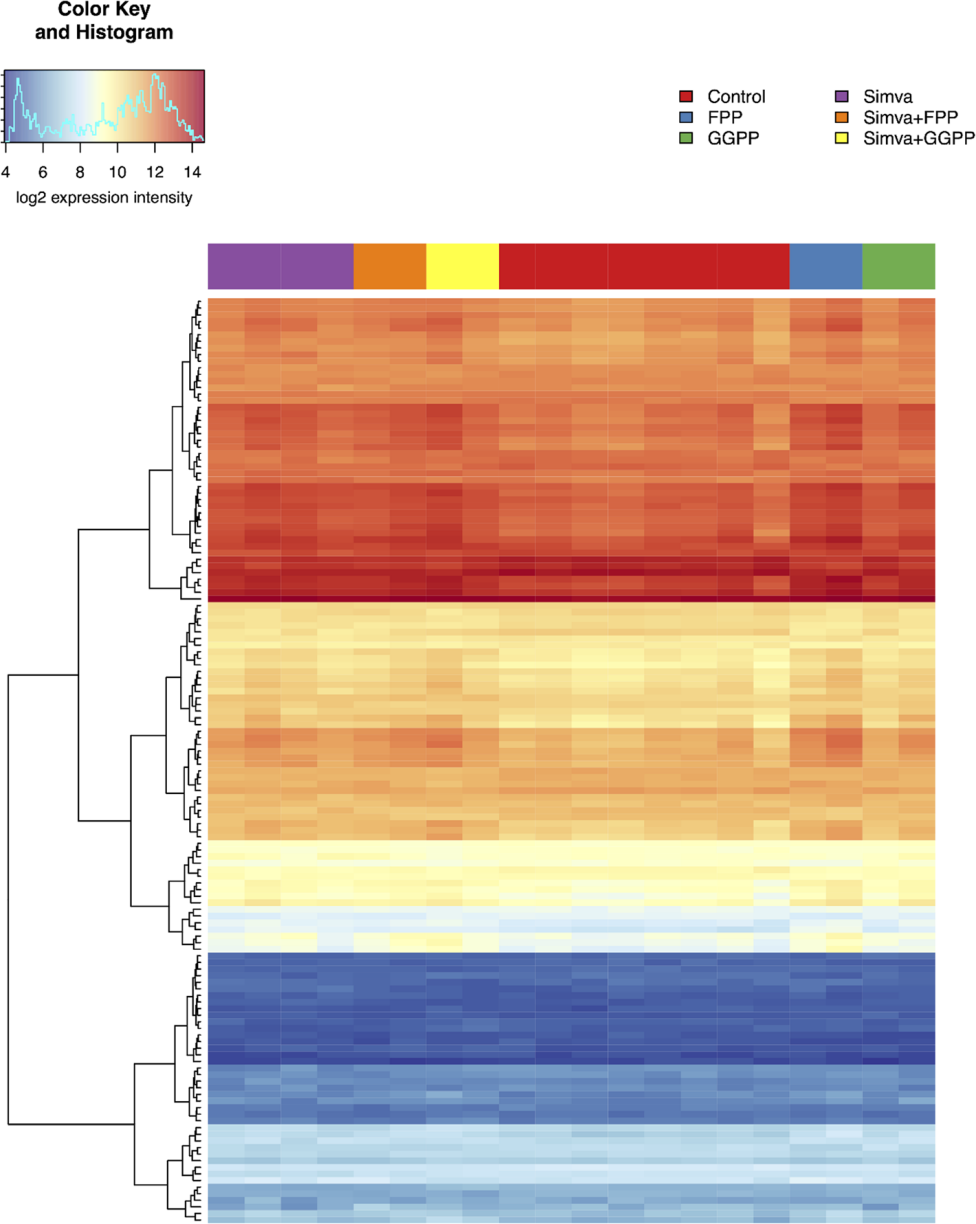
Heatmap illustrating log2-expression intensities of the genes that are involved in oxidative phosphorylation (KEGG pathway hsa00190). The list and sequence of the genes correspond to those stated in the Additional file 1: Table S4. Simva = simvastatin; FPP = farnesyl pyrophosphate; GGPP = geranylgeranyl pyrophosphate

There were eight carcinogenesis-related KEGG pathways significantly affected by simvastatin treatment (Table 2, see Additional file 3: Table S3 for all affected pathways). Deregulation of these pathways was eliminated by simultaneous treatment of the cells with simvastatin and FPP or GGPP. Only two pathways remained deregulated, namely the DNA replication and cell cycle. Simultaneous treatment of the MiaPaCa-2 cells with simvastatin and GGPP eliminated the effect of simvastatin on DNA replication and cell cycle regulation more prominently than simvastatin in combination with FPP, accounting for better efficacy of GGPP on cancer cell viability [8]. The expression of a large number of genes implicated in DNA replication, cell cycle regulation, and mismatch repair were affected by FPP alone (Table 2). While only marginally significant after treatment by simvastatin, the genes involved in oxidative phosphorylation were significantly upregulated by treatment with FPP, simvastatin and FPP, and simvastatin and GGPP (Table 2, Fig. 3).

**Table 2:** Signaling and metabolic pathways enriched by genes that change expression intensity after simvastatin, FPP, and GGPP treatment of MiaPaCa-2 human pancreatic cancer cells

KEGG Path ID	Path name	Simva	FPP	Simva + FPP	GGPP	Simva + GGPP
hsa03030	DNA replication	+	+	+	–	+
hsa04110	Cell cycle	+	+	+	–	+
hsa03430	Mismatch repair	+	+	–	–	–
hsa00100	Steroid biosynthesis	+	–	–	–	–
hsa03440	Homologous recombination	+	–	–	–	–
hsa04010	MAPK signaling pathway	+	–	–	–	–
hsa00240	Pyrimidine metabolism	+	–	–	–	–
hsa00190	Oxidative phosphorylation	(−)	+	+	–	+

Gene set enrichment analysis (GSEA) performed on KEGG signaling and metabolic pathways reveals the pathways enriched for genes that are differentially transcribed after simvastatin (12 μM) treatment, and the modulatory effects of FPP (17 μM) and GGPP (17 μM)

+ GSEA FDR < 0.001; − GSEA FDR > 0.001; (−) oxidative phosphorylation was only marginally significant in GSEA analysis by Tian with FDR < 0.05

*Simva* simvastatin, *FPP* farnesyl pyrophosphate, *GGPP* geranylgeranyl pyrophosphate, *FDR* false discovery rate

Quantitative PCR analysis of selected genes revealed in most of the gene tested satisfactory agreement with the microarray data obtained, thus supporting their validity (Additional file 1: Table S4).

## Discussion

Although prenylated proteins are estimated to constitute ~ 2% of the total mammalian proteome [19] (including key intracellular signaling proteins involved in carcinogenesis [20]), and many pre-clinical and clinical studies have implicated the role of protein prenylation in various human pathologies (such as certain cancers, cardiovascular, and/or neurodegenerative diseases), the therapeutic potential of isoprenoid synthesis inhibitors, including statins, still awaits to be explored in more detail [21]. In fact, various approaches to suppress K-Ras signaling are currently under investigation, although effective treatment is still far from the clinical application [10]. In this respect, it should be noted that statin use has been reported to significantly improve prognosis of pancreatic cancer in several [22–24], although not all clinical studies [25].

Consistent with this data, we previously observed that the inhibitory effects of statins on the viability of pancreatic cancer cells were partially prevented by concomitant addition of mevalonate, FPP, or GGPP, indicating the contribution of isoprenoids for growth and viability of pancreatic cancer cells [11]. Concurrently, Cafforio et al. have shown that the addition of mevalonate, GGPP, or FPP to cerivastatin-treated cells derived from human leukemia and myeloma resulted in complete recovery of both cell viability and proliferation; while no effect was observed by squalene supplementation [26]. However, other studies on the role of FPP and GGPP in the biology of various tumor-derived cells including cervical as well as head and neck squamous cell carcinomas [27], acute myeloid leukemia [28], or colon cancer [29] have produced different results. While in these studies FPP supplementation was shown to have had only a partial effect on statin-induced cancer cell apoptosis, that of GGPP was markedly higher. Even more so, in another in vitro study on human leukemia cells, inhibition of geranylgeranyl pyrophosphate synthase, leading to GGPP depletion with simultaneous FPP accumulation, led to enhanced statin-induced apoptosis of leukemia cells, while depletion of both isoprenoids resulted in the vanishing of the apoptotic effect [30] suggesting superiority of GGPP over FPP in the process of apoptosis in this particular model. A similar disparate effect was also demonstrated for colon cancer cells in a study by Agarwal et al., in which addition of GGPP prevented lovastatin-induced apoptosis, whereas FPP, even in high concentrations had no such effect [31]. These data apparently suggest different roles of both isoprenoids in carcinogenesis, and may also explain the discordant effects of these compounds on the gene expression profiles of the pancreatic cancer cells observed in our study.

We also demonstrated, that all the statins (except pravastatin) effectively eliminated the GFP-K-Ras protein trafficking to the cell membrane [11], suggesting that depletion of FPP caused by statin-mediated inhibition of the mevalonate pathway disables proper posttranslational modification and trafficking of the GFP-K-Ras protein. Our data extend previous observations by other researchers demonstrating the inhibitory effect of lovastatin on K-Ras protein translocation in mesothelioma [32] or NIH-3 T3 cells [33]. As proven in our current study, the localization of GFP-K-Ras protein has been effectively rescued with both FPP and GGPP supplementation, which is consistent with their rescue effect on the pancreatic cancer cell viability observed in our previous study [11]. The fact that both FPP and GGPP comparably rescued GFP-K-Ras protein trafficking to the cell plasma membrane (as suggested by Fig. 1) is likely to be the result of the cross-prenylation phenomenon [34]. The K-Ras protein preferentially undergoes farnesylation [34], but may also be geranylgeranylated, as demonstrated in cancer cells treated with farnesyltransferase inhibitors [35, 36].

Although both FPP and GGPP eliminated the block of GFP-K-Ras protein posttranslational modification caused by statins, the gene expression profiles after exposure of the simvastatin-treated cells to FPP and GGPP differed substantially. Genes whose expressions were most significantly affected by FPP treatment are largely identical with those affected by simvastatin treatment. This similarity was probably caused by the same inhibitory potential of FPP for HMG-CoA reductase, as reported for simvastatin [7, 37]. Nevertheless, this negative feedback was not observed for GGPP (Table 1), consistent with the fact that the excess of GGPP does not have an inhibitory effect on HMG-CoA reductase [38].

The observed data on gene expression in the pancreatic cancer cells treated with statin and isoprenoids might be explained by some complex regulatory activity, which FPP and GGPP exert on HMG-CoA reductase and other intracellular signaling pathways. These involve transcriptional (via modulation of sterol regulatory element-binding protein (SREBP) transcription factors), posttranscriptional (by accelerating protein acceleration, as described for statins), as well as posttranslational regulation [39]. In particular, changes could be seen in SREBP transcription factor regulated genes such as *MVD*, *FDFT1*, *HMGCS1*, and *LSS*, as well as RAS-related genes *RRAS*, *RAB17*, and *RHOB*. In addition, FPP and GGPP may directly (and specifically for each isoprenoid) influence the expression of Ras and Ras-related proteins [40]. Last but not least, both isoprenoids can directly modulate the activities of certain nuclear receptors. For instance, FPP can stimulate peroxisome proliferator-activated receptors (PPARs) [41], thyroid, estrogen, and glucocorticoid receptors [42], while GGPP itself has been shown to act as an antagonist of liver X receptor (LXR) [43]. Collectively, all this data demonstrates the multifaceted regulatory system exerted by isoprenoids. With a few exceptions, farnesylation and geranylgeranylation processes are highly substrate specific [19], further accounting for the observed changes in the gene expression profiles observed in our study.

**Fig. 4:**
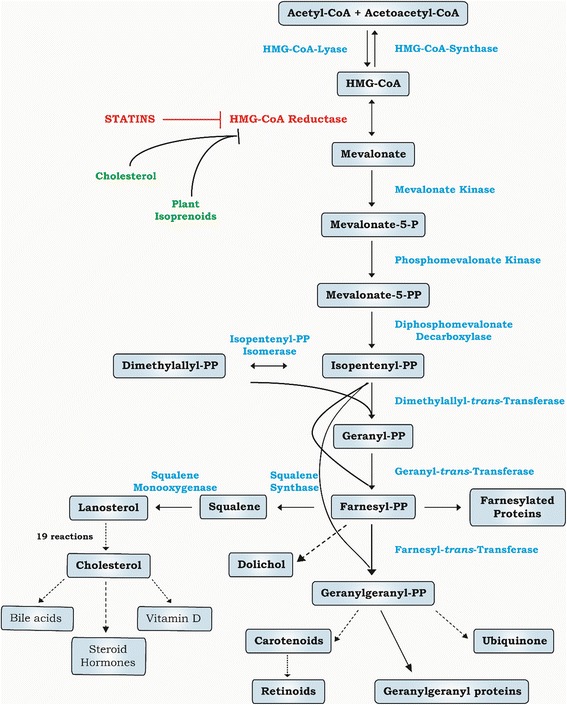
Scheme of the mevalonate pathway and isoprenoid synthesis. The mevalonate pathway (also known as the isoprenoid pathway) is an essential metabolic pathway in a human body producing a five-carbon building block called isopentenyl pyrophosphate (IPP). IPP is used to make fully functional biomolecules belonging into a large group of isoprenoids. Two intermediates in this pathway, FPP and GGPP, play also an immense role in post-translational modification of signaling proteins involved in a wide array of cellular functions including proliferation, and also carcinogenesis. The whole mevalonate pathway begins with acetyl-CoA, the rate limiting enzymatic step is catalyzed by HMG-CoA reductase (a therapeutic target of statins), whose inhibition leads to depletion signaling isoprenoids. Continuous and dashed lines represent one and multistep reaction, respectively. HMG-CoA reductase can be blocked by statins (in red)

We also observe an interesting effect of GGPP on those genes involved in the oxidative phosphorylation pathway. It is known that ubiquinone synthesis is blocked by the inhibitory action of statins [44], leading to the lack of ubiquinone in the mitochondria, with a consequent block of oxidative phosphorylation in the statin-treated cells. This, however, cannot be overcome by concomitant addition of GGPP or FPP, due to a lack of isopentenyl-PP (the FPP and GGPP upstream metabolites of the mevalonate pathway), which are also necessary for ubiquinone synthesis (Fig. 4). However, the results of the microarray analysis demonstrated that the process of the oxidative phosphorylation was only slightly affected by simvastatin, and was not affected by GGPP; however, the simultaneous treatment of cells with simvastatin and GGPP significantly changed the expression of genes encoding proteins of mitochondrial oxidative phosphorylation (Table 2, Fig. 3). In most of the cancer cell lines, the mitochondrial oxidative phosphorylation system is deficient, and the energy for growth (ATP production) is almost exclusively derived via glycolysis [45]. Whereas expressions of the genes involved in glycolysis were not affected in any of our experiments (data not shown), it seems that the anti-tumor effect of simvastatin on pancreatic cancer cells may also involve the inhibition of the mitochondrial oxidative phosphorylation, a phenomenon also described for simvastatin-treated prostate cancer cells [46].

One limitation of our study is high concentration of statins used. Since the clinical data on chemoadjuvant effects of clinically relevant doses of statins on pancreatic cancer are promising, it is apparent that further studies focused on determination of statins and isoprenoids in blood or even pancreatic cancer tissue might confirm our experimental data. In fact, such analytical methods are currently available [47] and are being used in our current studies [48]. Interestingly, gram doses of statins have already been used to treat cancer patients [49, 50], nevertheless it is certain that further clinical studies are needed to evaluate escalating doses of statins to define the therapeutic windows in oncology settings [51].

## Conclusions

Our data indicate that the anticancer effects of statins observed in numerous studies to a large extent are mediated through isoprenoid intermediates of the mevalonate pathway, as they influence expression of genes involved in multiple intracellular pathways.

## Additional files

Additional file 1: Table S1. List of primers used for quantitative real-time PCR analyses. **Table S4.** Quantitative RT PCR analysis of selected genes. (DOCX 18 kb)

Additional file 2: **Table S2.** Differentially expressed genes after simvastatin, FPP, or GGPP treatment of MiaPaCa-2 human pancreatic cancer cells. (XLS 1507 kb)

Additional file 3: **Table S3.** Gene set enrichment analysis on KEGG pathways affected by simvastatin, FPP, or GGPP treatment of MiaPaCa-2 human pancreatic cancer cells. (XLSX 22 kb)

## Abbreviations

FPP: farnesyl pyrophosphate
GFP: green fluorescent protein
GGPP: geranylgeranyl pyrophosphate
HMG-CoA: 3-hydroxy-3-methylglutaryl-coenzyme A
LXR: liver X receptor
PPARs: peroxisome proliferator-activated receptors
SREBP: sterol regulatory element-binding protein
