# Supplementary material for: Isoprenoids responsible for protein prenylation modulate the biological effects of statins on pancreatic cancer cells
Source: Lipids Health Dis. 2017 Dec 20;16:250. doi: 10.1186/s12944-017-0641-0 (PMC5738693; doi:10.1186/s12944-017-0641-0)
Supplement: Supplementary file 1 — List of primers used for quantitative real-time PCR analyses. Table S4. Quantitative RT PCR analysis of selected genes. (DOCX 18 kb) [file 12944_2017_641_MOESM1_ESM.docx]

**Table S1. List of primers used for quantitative real-time PCR analyses**

| **Gene** | **Left primer** | **Right primer** |
| --- | --- | --- |
| HMGCR | ctggggaattgtcacttatgg | gaggtcttgtaaattgatcttcgac |
| HMGCS1 | tgcattcaaacatagcaactga | cacctcacagagtatcttaatgttcc |
| KRAS | cactgttcacaaaggttttgtctc | ttggggagagtgaccatga |
| MCM2 | cagcagatcggagagaagga | aaggaatggcaccaatacca |
| MCM7 | agctacctgctgcactgctc | gctggtgcacataggtgatg |
| MVD | ttgtggagtggcagatgg | tgcctgtcagcttcttctca |
| ORC1L | aaggcctttgaagatgatgc | catctccagacagtgctgcta |
| POLA2 | ggccgaggagatcagtagttc | ggcgggtagagtgggtagt |
| PRIM1 | ctttgaagaatatgccttggttaat | tgtgagacttttggaagctttg |
| RFC3 | tgaagttcgtggaaggctgt | gctcatagtaagctgccatttg |
| Rhob | gcatgaacaggacttgacca | tgtgtcctccccaagtcagt |
| RRAS | ggcagatctggagtcacagc | acgttgagacgcagtttgg |
| TNFRSF10D | tcttacctcaaaggcatctgct | ccggaaaaggactctgtgc |

For explanation of gene symbols see Suppl. Tab. 2.

**Table S4. Quantitative RT PCR analysis of selected genes**

| **Gene** | **Method** | **FPP**  **vs. Control** | **GGPP**  **vs. Control** | **Simva**  **vs. Control** | **Simva+FPP**  **vs. Control** | **Simva+GPP**  **vs. Control** |
| --- | --- | --- | --- | --- | --- | --- |
| HMGCR | Microarray | 0.962 | -0.04 | **1.66***** | 0.625 | -0.134 |
|  | RTqPCR | -0.538 | 0.245 | 0.256 | 0.814 | **-1.42***** |
| HMGCS1 | Microarray | **1.2^+^** | 0.315 | **2.03***** | 0.762 | -0.191 |
|  | RTqPCR | -0.645 | 0.01 | 0.271 | 0.816 | **-1.36*** |
| KRAS | Microarray | **0.678^+^** | 0.058 | **1.45***** | 0.435 | 0.145 |
|  | RTqPCR | -0.454 | 0.228 | 0.192 | 0.462 | **-1.12**** |
| MCM2 | Microarray | **-0.725*** | -0.256 | **-1.22***** | -0.488 | -0.447 |
|  | RTqPCR | **-1.4*** | n.a. | -1.93 | n.a. | -0.623 |
| MCM7 | Microarray | **-0.955^+^** | 0.02 | **-1.26***** | -0.491 | -0.573 |
|  | RTqPCR | **-1.03**** | -0.984 | -0.887 | -0.569 | **-0.897^+^** |
| MVD | Microarray | **0.795*** | 0.126 | **1.1***** | 0.499 | -0.122 |
|  | RTqPCR | 1.21 | 1.25 | 1.21 | 0.382 | 0.787 |
| ORC1L | Microarray | **-0.825*** | -0.128 | **-1.36***** | -0.522 | -0.48 |
|  | RTqPCR | **-1.27*** | -1.39 | -1.25 | -0.576 | -1.4 |
| POLA2 | Microarray | **-0.651*** | -0.187 | **-1.08***** | -0.392 | -0.307 |
|  | RTqPCR | -1.32 | -1.22 | -1.19 | -0.379 | **-1.1**** |
| PRIM1 | Microarray | **-0.77*** | -0.156 | **-1.19***** | -0.404 | -0.005 |
|  | RTqPCR | -1.21 | -1.27 | -1.13 | -0.1 | **-0.968*** |
| RFC3 | Microarray | **-0.751^+^** | -0.105 | **-1.22***** | -0.533 | -0.645 |
|  | RTqPCR | **-1.1*** | -1.35 | -0.711 | 0.251 | -1.41 |
| Rhob | Microarray | **2.34*** | 0.203 | **3.45***** | 1.53 | 0.936 |
|  | RTqPCR | 1.73 | 2.32 | 1.93 | 2.1 | 1.12 |
| RRAS | Microarray | **0.899*** | 0.023 | **1.23***** | 0.375 | 0.451 |
|  | RTqPCR | 0.155 | 0.717 | 0.301 | 0.214 | 0.206 |
| TNFRSF10D | Microarray | 0.544 | 0.113 | **1.96***** | 0.458 | **1.64**** |
|  | RTqPCR | -0.01 | 1.12 | 1.09 | 0.275 | 2.05 |

Log-fold (base 2) expression changes in selected comparisons as detected in RT-qPCR and microarray analyses. Figures in bold denote statistically significant changes (p < 0.1 for RT-qPCR, and Storey's q < 0.1 for microarray data). The symbols denote: n.a. not available, **^+^** p or q < 0.1, * p < 0.05, ** p < 0.01, *** p < 0.001.

For explanation of gene symbols see Suppl. Tab. 2.

Simva = simvastatin; FPP = farnesyl pyrophosphate; GGPP = geranylgeranyl pyrophosphate
